# Better governance, better access: practising responsible data sharing in the METADAC governance infrastructure

**DOI:** 10.1186/s40246-018-0154-6

**Published:** 2018-04-26

**Authors:** Madeleine J. Murtagh, Mwenza T. Blell, Olly W. Butters, Lorraine Cowley, Edward S. Dove, Alissa Goodman, Rebecca L. Griggs, Alison Hall, Nina Hallowell, Meena Kumari, Massimo Mangino, Barbara Maughan, Melinda C. Mills, Joel T. Minion, Tom Murphy, Gillian Prior, Matthew Suderman, Susan M. Ring, Nina T. Rogers, Stephanie J. Roberts, Catherine Van der Straeten, Will Viney, Deborah Wiltshire, Andrew Wong, Neil Walker, Paul R. Burton

**Affiliations:** 1 Newcastle University, Newcastle upon Tyne, UK; 2 University of Cambridge, Cambridge, UK; 3 University of Edinburgh, Edinburgh, UK; 4 University College London Institute of Education, London, UK; 5 University of Bristol, Bristol, UK; 6 PHG Foundation, Cambridge, UK; 7 University of Oxford, Oxford, UK; 8 University of Essex, Colchester, UK; 9 King’s College London, London, UK; 10 NatCen, Brentwood, London, UK; 11 University College London, London, UK; 12 Imperial College London, London, UK; 13 Ghent University Hospital, Ghent, Belgium; 14 Goldsmiths, University of London, London, UK; 15 UK Data Archive, University of Essex, Colchester, UK; 16 NIHR Bioresource, Cambridge, UK

**Keywords:** Data ethics, Data governance, Data access, Data Access Committee (DAC), Governance, Participant involvement, Ethnography, Qualitative research, Interdisciplinarity

## Abstract

**Background:**

Genomic and biosocial research data about individuals is rapidly proliferating, bringing the potential for novel opportunities for data integration and use. The scale, pace and novelty of these applications raise a number of urgent sociotechnical, ethical and legal questions, including optimal methods of data storage, management and access. Although the open science movement advocates unfettered access to research data, many of the UK’s longitudinal cohort studies operate systems of *managed* data access, in which access is governed by legal and ethical agreements between stewards of research datasets and researchers wishing to make use of them. Amongst other things, these agreements aim to respect the reasonable expectations of the research participants who provided data and samples, as expressed in the consent process. Arguably, responsible data management and governance of data and sample use are foundational to the consent process in longitudinal studies and are an important source of trustworthiness in the eyes of those who contribute data to genomic and biosocial research.

**Methods:**

This paper presents an ethnographic case study exploring the foundational principles of a governance infrastructure for Managing Ethico-social, Technical and Administrative issues in Data ACcess (METADAC), which are operationalised through a committee known as the METADAC Access Committee. METADAC governs access to phenotype, genotype and ‘omic’ data and samples from five UK longitudinal studies.

**Findings:**

Using the example of METADAC, we argue that three key structural features are foundational for practising responsible data sharing: independence and transparency; interdisciplinarity; and participant-centric decision-making. We observe that the international research community is proactively working towards optimising the use of research data, integrating/linking these data with routine data generated by health and social care services and other administrative data services to improve the analysis, interpretation and utility of these data. The governance of these new complex data assemblages will require a range of expertise from across a number of domains and disciplines, including that of study participants. Human-mediated decision-making bodies will be central to ensuring achievable, reasoned and responsible decisions about the use of these data; the METADAC model described in this paper provides an example of how this could be realised.

## Introduction: managing access to complex and sensitive research resources

Genomic and biosocial research data about humans continue to proliferate, bringing with them questions about who should be able to store, hold and use which data and samples, for which purposes and with what safeguards. The research data landscape is changing with new forms of research data becoming available (e.g. next-generation sequencing, epigenetic and other ‘omics’ data [1, 2]) and existing data being increasingly accessible for research (e.g. via linkage of administrative, environmental or healthcare data to research data [3]). This complexity will only increase as new informatics technologies that enable citizen-generated data (e.g. social media, direct-to-consumer genetic testing and wearable sensors) become available to combine with research resources in novel ways.

### Open science and drivers for data sharing

Expectations surrounding how research data should be shared are based on international commitments to using, and re-using, publicly funded research and its outputs for the public good [4]. Data sharing is also supported by research funding agencies internationally through their policies on open science (https://www.nwo.nl/en/policies/open+science/data+management, http://www.allianzinitiative.de/en/archive/research-data/principles.html, [5–10]). Scientific benefits of data sharing are seen to include verification, replication and the ability to pool analyses, as well as potential cost savings [9–13]. Some of the drive for shared data use comes from patients and publics themselves: already, many rare disease patients advocate for greater sharing of genomic and clinical data; the quantified-self movement promotes and facilitates sharing of data from wearables [14, 15]; citizen scientists demand to access clinical and other randomised controlled trials data and have filed legal claims to realise those demands [16]. International policy positions research data and samples as a public good which can only be fully realised by their wide and appropriate use [17], though some types of research data such as that generated by commercial companies (e.g. pharmaceutical companies) sit outside this definition. Indeed, some have gone even further to argue that not sharing research data is a breach of participant, patient and public rights and is itself a harm [18]. While the open science movement advocates unfettered access to research practices and the data produced by them, when those data are individual-level human data, access and re-use must be managed within relevant legal and ethical requirements, taking account of specific agreements together with the reasonable expectations of the research participants who provided those data and samples. These requirements, agreements and expectations are embodied in the consents that study participants, or their guardians if they are minors, give at the outset of the study and, often, for new collections or sub-studies.

### Data sharing, longitudinal studies and the consent process

To date, the majority of genomic and biosocial research data made available for sharing in the UK have been derived originally from publicly funded research studies: longitudinal studies [19–21] (e.g. birth cohorts, household panel studies, other population and disease-based biobanks, clinical trials); survey and other social science datasets; and case-control studies, including case series collected/generated for research purposes and typically compared to a research-generated control group (e.g. Wellcome Trust Case Control Consortium [22]).

In longitudinal studies, the focus of this paper, it is not possible to foresee all possible research uses at the outset of a study. Data and samples may be used for long after their collection, sometimes decades afterwards. For studies whose *raison d’etre* is the provision of a scientific resource for which public good is the intended outcome and impact, renewed consent for each and every new research use would not only be unwieldy but would impede that aim. Instead, broad consent (within specific stipulations reflecting contemporary social values and scientific practice) are sought for future uses of the data and samples collected. In the UK, under the Human Tissue Act 2004 (https://www.legislation.gov.uk/ukpga/2004/30/contents) and Health Research Authority, the principles and contents of information given to study participants in consent and participant information documentation for different forms of data and samples are prescribed [23, 24].

But consent is a process, one which does not finish with the receipt of information and the signing of a form. Ethical approval for studies using broad consent includes mechanisms to ensure that those consents are respected and the expectations inherent in them are maintained, for example, explicitly stating which bodies can approve data and sample access. Appropriate, responsible data management and governance of the uses of those data and samples are foundational to this ongoing consent process in longitudinal studies and an important source of their trustworthiness in the eyes of individuals who have contributed data and continue to permit its storage and use. While trust is crucial for ensuring public support for research generally, for longitudinal studies, this requirement is particularly acute as ongoing research participation is vital to the longevity, productivity and impact of those studies. Research participants quite reasonably expect that their privacy (including confidentiality) will be protected and that uses of the data and samples they contribute will fall within agreed and anticipated parameters.

### Data Access Committees

While Research Ethics Committees (RECs, also called Institutional Review Boards or Research Ethics Boards) interpret the ethical, legal and social frameworks within which studies are conducted and determine how data and samples may be used, data access permissions in the UK are governed by a hierarchy of regulatory processes and bodies depending on the data’s sensitivity and potential disclosivity (the ease and precision with which individuals may be identified). Light-touch administrative processes manage low-dimensional data with a low risk of disclosure or other participant harms. Data Access Committees (DACs) manage more complex or higher-risk data access. Key assessment criteria for determining the release—and access route—of such data include (1) consistency with original ethical approval, including consents and information materials and the stated aim of the data collection, and the risk of identification of individual participants; (2) issues that may directly concern individual participants, such as the risk of identifying a previously unknown disease or strong disease determinant which may warrant clinical action, or the risk of bringing a long-standing study into disrepute; (3) unreasonably damaging the intellectual property of the project, or otherwise undermining the effort invested by its investigators or data custodians (often more than one generation of researchers).

DACs may consider any risks which may impact the individual participant or otherwise breach their expectations, thereby provoking them to withdraw from a study. In addition to the above criteria, some DACs assess the quality of the science or potential public benefit for all applications, and nearly all committees assess the quality and potential benefit of the science when there is a request for use of a finite resource (e.g. blood samples). Across the research/scientific community in the genomic, health and social sciences, there is a strong consensus that the application of all such criteria (when applied in a proportionate and transparent manner) is good for society *and* ‘good for science’ [25].

### Data access governance in the UK

In the UK, access to data and samples—the ‘resources’ of longitudinal and other research studies—is operationalised by a networked series of independent data repositories and independent data governance infrastructures, though some longitudinal studies successfully operate in-house or partially in-house governance and data issue mechanisms. Governance of access permissions (ethics and policy oversight) and governance of data issue (technical governance) may be managed jointly or by separate infrastructures. Each of these governance infrastructures is designed to ethically, legally, efficiently and securely manage access (permissions and/or distribution) to research resources of varied levels of complexity and sensitivity. For many UK longitudinal studies, data can be accessed online by bona fide researchers and are governed by end-user licences. Access decisions may also be made based on algorithms and straightforward rules for legitimate access. These are generally rapid mechanisms for access, notwithstanding any additional time required for online training upon registration to a data repository (e.g. UKDA training in data management [24]) or additional administrative processes for managing cost recovery. The data released by these mechanisms are those for which there is deemed to be a low risk of individual disclosure and which are considered not to raise additional ethical issues. Some data will only ever be made available in secure privacy-protected settings—where researchers travel to the data location (e.g. secure air-gapped data centres) or send the analysis to the data (e.g. DataSHIELD) and receive back only the analytical outcomes [26]. Use of finite resources such as blood, urine or other biological samples will always require additional oversight for legal (e.g. Human Tissue Act 2004 requirements), ethical and scientific reasons and because each use of samples necessarily precludes future uses of those samples and therefore must be judged more carefully.

Classes of research data which are potentially sensitive, insofar as they are disclosive and/or raise particular ethical concerns (e.g. incidental/secondary findings), require additional access oversight. Beyond the character of the data themselves, some research uses of data are also potentially controversial and may thereby raise ethical issues and potential harms for research participants themselves, or for the longitudinal studies of which they are a part. Some types of research are particularly likely to raise issues of potential participant and/or study harms, e.g. those involving potentially stigmatising issues, such as mental health, sexuality, criminality and certain diseases, or those in which researchers are perceived to make a commercial gain. Particular forms of or combinations of data also raise risks of disclosure. That individuals can be identified within a genetic dataset has been demonstrated methodologically [27, 28], but only where there is a reference sample/sequence available [29]. For most practical applications identification is unlikely, though this may change with increasing availability of commercial genotyping. Identification of individuals via phenotype data, though less certain than genetic identification, is easier to enact if large numbers or certain classes of variables are combined in analyses (e.g. place of residence if defined too narrowly, minority ethnicity, disability, rare disease status) or, as in longitudinal studies, repeated measures are available. Research questions which further combine genotypic and phenotypic data and/or samples may increase the potential for disclosure of individual identity. Whether the important issue for disclosure is one of identifying the individual per se (i.e. that a particular individual belongs to a longitudinal study) or whether it is about identifying the individual *and* information about them is an empirical question that requires further examination. Where there are higher risks of disclosure or the proposed research raises other potential harms, online registration and end-user licencing are not sufficient safeguards. Decisions about complex societal values are ultimately not amenable to a simple algorithmic approach whether derived heuristically or, for example, via machine learning. We argue that a human-mediated review and decision-making process are then required. Moreover, we argue that study participants must be central to this decision-making, seeing through to its conclusion the ongoing consent dialogue initiated in consent and participant information, procedures and documents.

## Methods

In this paper, we present an ethnographic case study [30] exploring the foundational principles of one infrastructure for Managing Ethico-social, Technical and Administrative issues in Data ACcess (METADAC), i.e. the ethics and policy oversight of data access. The METADAC infrastructure (that is, the METADAC Access Committee and associated work of the pilot project described below) aims to put into practice the tenets of responsible data sharing [31, 32] and, in particular, operationalises incorporation of study participant voices and perspectives in access decision-making. The case study approach allows an in-depth consideration of a particular instance of practice, or ‘case’. As Stake [33] describes, the case study is ‘the study of the particularity and complexity of a single case, coming to understand its activity within important circumstances’ (p. 6). Case studies may involve a range of methods, the prime criteria being those most able to elicit in-depth, rather than summary, understandings of the object of study; in this case qualitative methods. The analysis in this case study here was informed by a qualitative ethnographic evaluation study conducted alongside the METADAC pilot. The ethnographic evaluation included observations of METADAC committee meetings (*n* = 16) and policy development workshops (*n* = 4) from June 2016 to October 2017. Meetings were observed by JTM. Twenty-seven interviews were conducted by JTM from March to August 2017 with 18 internal METADAC stakeholders (committee members, funders, study representations, technical review team members) and 9 METADAC applicants (from 31 invitations). Internal stakeholders were asked about METADAC processes and policies. Applicants were asked about their experience of the application process. Meeting observations and interview transcripts were coded thematically using the constant comparative approach [34] and following the steps provided by Braun and Clarke [35] for organising analysis without the imposition of a specific epistemic framework. This approach involves researchers familiarising themselves with the data corpus (e.g. the interview data in this study), generating initial codes and then developing, reviewing and defining themes. Having identified our themes using this approach, we took a constructivist-interpretivist approach to interpret the themes, i.e. ‘particular actors, in particular places, at particular times, fashion meaning out of events and phenomena through prolonged, complex processes of social interaction involving history, language and action’ and ‘that to understand this world of meaning one must interpret it’ [36]. We present this analysis with representative extracts from the interviews and description derived from the observational notes of the meetings.

## Results: the METADAC infrastructure

The METADAC infrastructure hosts a Data Access Committee (the METADAC Access Committee) governing access to the complex and sensitive genotypic and phenotypic classes of data and biological samples (see Table 1) generated by five longitudinal studies in the UK [37]: the National Child Development Study (a.k.a. 1958 Birth Cohort) [38], 1970 Birth Cohort Study (BCS70) [39], English Longitudinal Study of Ageing (ELSA) [40], Millennium Study [41] and *Understanding Society* (UK Household Longitudinal study) [42, 43]. It also acts as an appeals committee to one other longitudinal study (TwinsUK). METADAC has become the UK’s only independent, multi-study data and sample access infrastructure for longitudinal studies. The 3-year METADAC pilot brought together, under one infrastructure, a number of separate data and sample access structures. Meetings to review applications are held at six-weekly intervals, primarily as teleconferences and twice annually through face-to-face meetings, when other planning and policy development workshops also occur. Administratively, the METADAC Secretariat manages logs and performs a quality audit of all incoming applications. The Secretariat serves as the hub for all METADAC communication and interactions with and between stakeholders. Three key structural features provide the foundation for practising responsible data sharing: independence and transparency; interdisciplinarity; and participant-centric decision-making. These features are realised in the access assessment criteria by which the METADAC Access Committee makes its decisions (see Table 2).

**Table 1:** Classes of data and their access regulation

	Data type and sensitivity	Data available from	Governance	Data distribution
Class 1	Low risk survey/phenotype-only data, e.g. social, economic, psychological and health data.	UK Data Archive	Requires registered access—online (clickable) end-user licences.	Data is distributed by the UK Data Archive.
Class 2	Low sensitivity genetic-only data, e.g. genome-wide single nucleotide polymorphism [SNP] data generated from a standard chip with no phenotype information except gender and a coarse measure of area of residence.	European Genome-phenome Archive (EGA)	Requires application to the Sanger DAC, administrative decision based on algorithm of criteria for access.	Data are distributed by the EGA.
Class 3	Potentially disclosive survey/phenotype-only data, e.g. small geographic area. Requires application to a study-based Data Access Committee made up of study investigators and study technical staff.	Study DAC	Data may be issued on special licence or may be accessible only in on or off-site data safe havens.	Data is distributed, or the data safe haven hosted, by the study.
Class 4	New forms of genetic-only data, e.g. exome sequence and epigenetic data, are reviewed by METADAC until their ethical implication/sensitivity is established.	METADAC	Application requires review and approval from the METADAC Access Committee. Application includes signed agreement to conditions of use. Data use is monitored annually.	Data are distributed by the EGA.
Class 5	Genetic-only data with known ethical issues, e.g. incidental findings risk in exome data.	METADAC	Application requires review and approval from the METADAC Access Committee. Application includes signed agreement to conditions of use. Data use is monitored annually.	Data are distributed by the EGA.
Class 6	Biological samples. Use of biological samples will always require additional oversight for ethical and scientific reasons.	METADAC	Application requires independent scientific review and approval from the METADAC Access Committee.	Samples are issued by the relevant study under Material Transfer Agreements.
Class 7	Any combination of Classes 1 to 6, e.g. individual-level phenotype data linked to genotype data or samples, is potentially more disclosive than any one class of data alone.	METADAC	Application requires review and approval from the METADAC Access Committee. Application includes signed agreement to conditions of use. Data use is monitored annually.	Combined datasets are issued on unique IDs and distributed by the study and EGA.
Class 8	Non-research data. High risk when multiple variables are combined.	Various	Certain administrative datasets, e.g. education and criminal records, are available for research via the Administrative Data Research Network. Individual-level health data is available for research via NHS Digital).	Various

**Table 2:** METADAC assessment criteria

1. The application has been submitted by bona fide researchers (using the MRC definition [21]). 2. The application does not risk producing information that may allow individual study participants to be identified. 3. The application does not violate (or potentially violate) any of the ethical permissions granted to the study and/or any of the consent forms signed by the participants or their guardians. 4. The application addresses topics that fall within the acknowledged remit of the project, as understood by participants. 5. There is no significant risk that the application might upset or alienate study members or of reducing their willingness to continue as active participants. 6. There is no significant risk that the application might harm individuals in the study, or the study as a whole. 7. The application does not require access to a depletable finite resource (whole blood extracted DNA, blood, saliva and urine). OR The quality of the science has been reviewed formally by independent external reviewers (agreed by METADAC) and has been judged to merit the use of finite biological samples. NB: Applications for finite bio-samples are seen as being in competition with other potential future uses of those samples, and therefore the quality of the science is reviewed formally. 8. Includes a high quality plain language summary—following METADAC guidance.	

## Independence and transparency

One way of safeguarding the right to privacy and the right to benefit from science is to ensure a robust and independent data access process, especially for the most complex and sensitive research resources. The METADAC process has promoted fair, consistent and transparent practices of data access, including making these openly available on its website. An independent mechanism for access, particularly for more complex and sensitive data and sample access, supports these processes. The importance of independence is recognised by funders, for example, Wellcome’s recent Longitudinal Population Studies Strategy [8], and reflected in its criteria for funding longitudinal studies:

If it is not possible to fully inform participants about how their data could be used, it is essential that robust governance is in place, for example, ensuring there is an independent, accountable decision-making body that can provide assurances that data are being used and linked appropriately and responsibly. (p.6).

Four additional benefits of independent, multi-study access processes are clear and go beyond the values and norms of fair, transparent and ethical practice. First, in the competitive academic environment in which most researchers work, data ‘hugging’ or hoarding—real or perceived—by researchers internal to a study can result in constraints on sharing [44–46]. Independent access processes, be they via registered access [47] or DACs, support data accessibility. Second, study-independent, multi-study access bodies can facilitate data discovery. Year-on-year increases in applications and approvals through METADAC (a 2.5-fold increase over the first 24 months of operation) are a testament to the increasing international interest in the use of UK longitudinal studies and demonstrate the role of METADAC in enabling access to study resources. Moreover, researchers applying to METADAC are signposted to other similar studies and are able to make data and/or sample access applications to multiple studies simultaneously. Third, an independent committee allows determinations to be made about new uses of data and samples out-with the natural tendency of studies to be risk-averse regarding their own resource. As users of the resources return derived datasets and code for variable creation, rapid assessment of the ethical/governance implications of the uses and governance of new forms of data (e.g. exome sequencing and epigenetic data) is actioned, and the parameters for onward use are established through consensus. But of course, independent does not mean detached. Having representatives from the studies at the table is vital and enables the Access Committee to ensure that the particularities of the study are taken into account and that their future uses are optimised. METADAC has a responsibility to the ongoing ‘health and wellbeing’ of the studies and their participants and not simply to facilitate the exploitation of their data and samples. Fourth, METADAC enables learning across the studies under its remit, sharing good practice but also providing leverage to change practice. For example, one of the studies under METADAC governance, Understanding Society (the UK Household Longitudinal study), has been able to extend the availability of its genetic data resource internationally. The governance of multiple studies made it evident that Understanding Society’s historical restrictions on international access to its data were not a fundamental feature of longitudinal studies in the UK—other studies had no such restrictions. The existence of international access practices in the other studies under METADAC enabled the study directors to reassure their stakeholders that extending their access practices to harmonise with those of others was safe, proportionate and ethically acceptable. In short, there has been a virtuous circle of learning amongst METADAC studies.

## Interdisciplinarity

Because no individual or group can possibly hold knowledge of all aspects of research, research ethics and governance [48], the METADAC Access Committee is comprised of committee members with social, biomedical, ethical, legal and clinical expertise and individuals with study-facing expertise. Committee meetings also include, as observers, study PIs or study representatives, funder representatives and members of the Technical Review Team (TRT) who support the decision-making. The METADAC Secretariat and the ethnographic observer (JTM) also attend meetings. Where appropriate, the committee also takes advantage of third-party specialist knowledge, particularly where an applicant seeks to use finite samples. The METADAC Access Committee is chaired by a social scientist with ethics expertise (MJM), and its deputy chair (NW) has bioscience and bioinformatics expertise. The TRT is chaired (PRB) by a biomedical scientist with data science expertise.

The ethnographic study demonstrated that each of the contributors to the METADAC Committee brings particular understandings and perspectives which contribute richness to collaborative decision-making but that these are understandings that are not rigidly bound, with many members contributing across these domains.

Study-facing committee members, currently two members of longitudinal studies not governed by METADAC, bring (i) embedded and embodied subjective experience of longitudinal studies and thereby of study participants’ reasonable expectations, and (ii) insights into the project proposals that are not coloured by specialist knowledge of the same field. While researchers may be carried along by the importance and interest of a particular exploration (it answers an interesting research question), study-facing members are more likely to see each application ‘anew’ and spot where concerns may be raised for participants. We elaborate this contribution further below.

Committee members with legal and ethical expertise bring (i) knowledge of relevant laws and regulation (particularly data protection law, access, consent and issues around data privacy and biomedical research); (ii) understanding of the ethical norms, values and principles underpinning decision-making and their implications for protecting and promoting the rights, interests and welfare of participants, researchers and the broader community; and (iii) awareness of the remit and purpose of DACs as well as ensuring the clarity and transparency of METADAC practices and the documentation which seeks to explain them.

Biomedical scientists bring (i) an awareness of methodology, used not to judge the science but instead to understand whether variables requested are ‘necessary and reasonable’; (ii) understanding of the biomedical background to research, to judge the likely potential for incidental or secondary findings in research which at first glance may not raise clinically actionable findings; and (iii) awareness of related published material or similar data resources that may assist applicants in carrying out research.

Social scientists bring (i) methodological expertise to assess the aims, feasibility and study designs being proposed; (ii) understanding of social and environmental contexts when and where data were collected and their potential impact; (iii) awareness of issues that may give rise to ethical or public concern, often reframing scientific descriptions which uncover simplistic or paternalistic assumptions which may trigger participant concerns; and (iv) experience of working with other epidemiological and longitudinal studies.

Clinicians bring understanding of (i) both the seriousness and treatability of genetically influenced illnesses; (ii) the sometimes blurred boundaries between research and clinical practice, highlighting the complexities and impact of communicating uncertainties to patients and their families, especially in the contexts of interpreting genetic variants and reporting unlooked for findings; and (iii) a working appreciation of negotiating meaningful informed consent to genetic testing with patients when results may not be predicted or imminent.

The TRT provides practical insight on the affordances of the studies and a broad evaluation of disclosure risk and incidental finding risk, based on a knowledge of the size of the dataset, prevalence and heritability of the condition and issues that will require further detailed discussion in committee. The TRT includes individuals with expertise about the study data and other resources. They offer the Access Committee insight into what data resources are available, the format and affordances of those resources and, for example, whether there are specific technical or other restrictions on their use.

Study PIs/representatives bring a strong awareness of the field generally and their own studies in particular; the availability and relevance of data, any weaknesses in the data (e.g. a low response rate in a particular questionnaire) and other relevant data not known to the applicant—whether from a new wave or from another longitudinal study with similar data. Similarly, funders contribute understanding of strategic and policy direction nationally and internationally. In practice, any member may bring a contribution from many of these types.

This rich mix of participants brings to bear perspectives and understandings which support independent and transparent decision-making. During the ethnographic evaluation that has run in parallel to the formation of METADAC, internal stakeholders have frequently highlighted the value of having a range of disciplinary expertise in the Access Committee:

A multi-disciplinary approach is really vital so that we have both the technical expertise, ethical expertise, social sciences expertise, so that all those elements can be brought to one place to determine what is the optimal approach. (from evaluation interview with METADAC Committee member)

If there are ethical issues, there is someone who is a specialist in that and who’s thinking about it and brings issues up if they need to be discussed …there are lots of different people on [METADAC] from lots of different backgrounds and disciplines ... So there are different people thinking about these things. (from evaluation interview with METADAC Technical Review Team member)

In the ethnographic evaluation, the diversity of the committee was seen to produce higher quality reflection and analysis on matters of concern [49]. Participants described that hearing perspectives from other fields improved their own critical thinking. But, as Fitzgerald and Callard [50] put it: ‘There are two current certainties in … interdisciplinary research: everyone wants to do it, and no one quite knows how’ (p.23). While the benefits of multi-, inter- or transdisciplinary working in data governance are still emerging and deserve careful documentation, for METADAC, there appears to be some clear benefits, particularly in supporting a rounded approach that takes account of a plurality of perspectives in coming to consensual decisions. That is not to suggest that consensus is necessarily or always straightforward; it requires work. It is not simply multiple perspectives from distinct disciplines that are brought to bear in decision-making (not least because some members already span disciplines), but also multiple standpoints or subject positions [51, 52]; more colloquially, we say people ‘wear many hats’. Individuals taking part in METADAC decision-making inhabit and draw upon multiple subject positions: researcher/researched, data custodian/data producer, etc. We argue that it is these multiple subject positions which are important in decision-making precisely because they call upon the different interests, commitments and expectations which are central to ensuring decisions made about data and sample access are coherent with, and respect the contributions made by, study participants.

## Discussion: participant-centred decision-making

Engaging the perspectives of stakeholder communities within research and involving them in decision making are lauded for meeting ethical expectations and norms as well as for its (potential) pragmatic outcomes, improving the alignment of research with societal values and the relevance of research outputs or their translation [48]. Engagement is variously understood as a form of democracy, an act of respect and an acknowledgement of human rights [53–59]. In Braidotti’s vision of affirmative ethics [60, 61], the understanding that ‘we are all in this together’ even if we are ‘not one and the same’ [62] places upon science an injunction to work *with* the communities, public and research participants who are both the basis and beneficiaries of science. Of course, study participants are already at the centre of longitudinal studies; it is only with their permission and with their contributions of data and samples that the studies endure. Respecting those contributions means that participant-centredness must do more than simply consider them research *subjects*. Engaging study participants in meaningful decision-making is both an ethical and practical solution, enabling alignment with participant expectations, social norms and values and, arguably, improving study outputs as a result. As we argue elsewhere, engagement brings study participants closer to the research but also brings the research and researchers closer to them [48]. In the case of longitudinal studies, there is a long history of study participant involvement in decision-making. The Avon Longitudinal Study of Parents and Children (ALSPAC) has an advisory panel comprised of study participants and has included on its ethics committee original cohort participants (now nearing 26 years of age) for the past decade and parent members for much longer [63]. ALSPAC study participant members now number half of the ethics committee and their involvement in ethical decision-making is normalised and embedded. In METADAC, the need for multiple perspectives, met through the interdisciplinary constitution of its Access Committee, demands the inclusion of study-facing participants. By incorporating study-facing members, METADAC was seen by members and observers of the committee as contributing new and valuable voices to discussions about data access and use and expanding how Data Access Committees have historically functioned:

They’re the ones who have insights, that nobody else has, on the anxieties, the concerns, the hopes about how their data will be used, so they absolutely need to be represented. (from evaluation interview with METADAC Committee member)

Participant-centredness can, of course, take many forms: it can be agonistic or it can be solidaristic; it can be prescribed or it can be negotiated [64]. In scientific contexts, participatory activities can range from unidirectional communication and outreach, through various levels of involvement in decision-making, to participant control, as in citizen science endeavours. Each has advantages and limitations. In METADAC, which requires understanding and expertise from many perspectives in order to make responsible decisions, participant-centredness takes three forms: (1) respecting study participant expectations, (2) involving study participants in decision-making roles and (3) communicating the results of access decisions to participants (and others) in a format that is clear and accessible, for example, in plain language summaries.

### Study participant expectations

Participant expectations of longitudinal research are central to METADAC’s access decisions. Criterion 5 (Table 2) seeks to ensure that there is no significant risk of *upsetting or alienating* participants. While this is an instrumental value, in that it seeks to reduce loss to a study or studies, it is also primarily a value-based position aimed at respecting the participation and on-going commitment of study participants. Though in a cognate context, Caldicott’s injunction that participants (in her report, patients) should not be ‘surprised’ by the uses of their data and samples also holds true for METADAC. Assessment criterion 5 (Table 2) is called upon in METADAC Access Committee meetings in the form of two questions: (1) Would the research proposed by an applicant be likely to upset or alienate participants? and (2) Would the research breach the reasonable expectations of participants? It is via these questions that issues of potential sensitivity and controversy may be addressed. It might be plausible to construct an algorithm to identify certain classes of research as sensitive, and indeed for one of the studies under METADAC oversight, there is a specific list of research topics and types which are restricted. Specifically, the 1958 Birth Cohort explicitly restricts studies of the genetics of intelligence, sexuality and criminality. This stipulation is based on information provided to nurses during their formal training for taking samples from study participants. While not included in the information material given to study participants during consent, which are always the foundation of legitimate access requests, a previous committee determined that nurses might reasonably have been expected to communicate those exceptions to at least some study participants, and therefore, these continue to be respected. A list of restricted variables has been identified, but decisions about how and whether the research purposes proposed for their use are acceptable and approval granted are often more complicated than simple yes/no determinations. A level of interpretation and judgement is necessary, one which requires human decision-making.

### Study participant involvement in decision-making

An ethnographic (observational) study of a predecessor to the METADAC Committee identified, during access decisions, repeated consideration of what the participant or public might ‘think’ of a particular application for data use [44]. In that DAC, however, understanding of participant’s voice was hypothetical only, because no participants or other study-facing members were involved in DAC decision-making and no empirical account of their views was available (e.g. in qualitative studies of their expectations of involvement). The METADAC infrastructure addresses this absence by including two study-facing members who are themselves participants of longitudinal studies. To avoid conflicts of interest, these study-facing members are independent of the studies for which METADAC provides oversight. This decision was taken to avoid the assumption that study-facing members directly represent the study population. While two committee members cannot feasibly represent the individual interests of tens of thousands of study participants, the aim is to ensure that the embodied, material and emotional experience of being a study participant is incorporated into data governance decision-making. In this pilot phase of METADAC, development of study-facing members’ involvement is assisted by involving individual study participants who also happen to have active research careers in the social sciences.

METADAC has avoided treating study-facing members’ views as ‘non-expert’. Involvement of study participants in decision-making roles requires active work to ensure it is meaningful; a learning process that means that the contributions of these study participant members deepen as their knowledge and understanding of governance issues grow. Pre-meetings are held with all new members of the METADAC, whatever their background, and continue for study-facing members as often as they wish, to create a space for longer discussion of applications and other issues that is typically available in a 90-min committee meeting. After each committee meeting, informal debrief sessions are hosted to make sure that any uncertainties are resolved, wider observations can be aired and suggestions made. Active chairing in committee meetings and an inclusive culture facilitates all committee members to express their positions. The Secretariat acts as the first point of call when resolving practical problems, and the Access Committee as a whole can be used to develop insights into particular areas of procedure or policy.

Even within the acknowledged limitations above, decisions made with study-facing members create different outcomes to those made without their insight and experience. For example, the embodied sense of discomfort or unease from the participant perspective (or indeed from other members) warrants greater scrutiny of applications by the Access Committee and can and does encourage different decisions to be made. Some areas of sensitivity are not easily exposed through schematic, algorithmic or technical evaluation. We call upon study-facing members to scrutinise areas of uncertainty, along with other members, to articulate a range of potential study participants’ responses in order to highlight potential risks and harms of any given application and to anticipate how even well-intentioned research outcomes can produce harms, including those caused by sensationalised media reactions. Some of this reasoning can be messy and speculative; it stays with the troubling or unforeseen parts of an application and tries to consider how the expectations of science may align with or compromise those of study participants. In this regard, study-facing members bring a unique perspective and position to the committee. But they do more than this. In the METADAC, the study-facing members have been central in developing policy, particularly as new forms of data become available. Deep and often lively discussions during METADAC development workshops become the basis of policy and processes (e.g. recent access policy developed for exome sequence data and epigenetic data). In this way, decision-making in METADAC occurs *with* study participants as part of an ongoing, collaborative and negotiated process.

### Plain language summaries

Although plain language summaries (PLSs), or ‘lay summaries’, are an expectation of communication of much research—and most funders now require these for their applications—researchers often do not communicate their work in ways which are clear or accessible to non-expert audiences. METADAC owes a duty to communicate the outputs of their contributions to the participants of those studies for which it provides oversight and to their funders. PLSs are published on the METADAC website [1]. Development of PLS policy, processes and guidance, therefore, has been taken seriously, and METADAC’s study-facing committee members assess each PLS according to guidance provided to applicants [65]. As a reflection of the care by which PLSs are reviewed, and to ensure data use is transparent to study participants and the wider community, many applicants are asked to improve their PLS. While guidance is readily available, time-poor researchers do not always fully absorb the instructions or do not recognise the importance attached to PLSs by METADAC. The chief reason applicants are asked to revise their applications is on the basis of inadequate descriptions of their research.

### Limitations

This ethnographic case study was informed by the evaluation of the METADAC pilot. As such, it represents findings at an interim stage of METADAC’s development and should be read with this limitation in mind. The ethnographic methods used here provide a deep understanding of a phenomenon. Though they are not statistically generalizable, the findings, as expected of qualitative studies employing quality criteria for rigour and trustworthiness, can be considered theoretically generalizable to cognate settings.

## Conclusions: future considerations for data and samples access

At present, there is a strong strategic investment in ensuring not only the optimisation of research data use, but also of routine data generated by health and social care services and other administrative data services. Moving forward, the international research community is proactively working towards creating a world in which all such sources of data can, where beneficial, be integrated, linked and jointly interpreted; this potential has been demonstrated in the UK in projects such as The Farr Institute, the Administrative Data Research Network, Connected Health Cities, the pan-London Health Information Exchange and the Medical Research Council’s Health Data Research UK initiative [3, 66–69]. The aims of such initiatives and the enhanced data utility they provide are three-fold: (1) to better use data to directly inform front-line clinical care and public health, (2) to enhance the evidence base for health and social care planning and strategic development and formally evaluate key initiatives in these domains and (3) to enhance the quality and scope of research in health science, bioscience and the social sciences. Given the desirability of such aims and current investment by governments and funders, it is inevitable that over the next decade, DACs are going to have to deal with increasingly large volumes of data generated from the health, social care and other administrative data sources.

At least some DACs in the future are going to have to be able to deal simultaneously, and effectively, with the data access criteria they already apply to research data *and* the laws, rules and regulations applicable, in the UK, to National Health Service (NHS), social care and administrative data (e.g. education, criminal records, national statistics). Whether research DACs evolve to deal with NHS data (or as gatekeepers for NHS data), taking account of the real risk of identification of at least some individuals, even after pseudonymisation and similar privacy-protecting mechanisms, will demand a profound change in perspective. In addition, governing these data will create a substantive additional workload and a need to rapidly explore and develop appropriate systems shortcuts and technological solutions to some aspects of the assessment. Empirical evidence demonstrates that DACs face serious challenges when a new class of data suddenly becomes available: part of the argument for creating METADAC was that a predecessor DAC was not set up and therefore not able to deal effectively with newly available genomic data. METADAC and several studies under it have had to work hard (and rapidly) to modify the systems and structures for releasing conventional SNP-based genome-wide association data to deal with full DNA sequence data and with methylation data—even though many of the principles are unaltered. Other studies, not part of METADAC, have invested extensive time in considering how—if at all—to manage and govern new data classes such as data generated via social media or image data.

The evolution of DACs that can deal jointly and effectively with all of the criteria and rules applying to routinely collected health, social care or administrative data and all of those already applied in dealing with research data will be an equivalent challenge but of a much higher order. This will particularly prove to be the case when DACs have to start considering data generated and interpreted in real time; indeed, many new applications of health data in the future will require that research data from individuals can be combined with real-time health service data to enable rapid clinical responses for diagnosis or management. If both the research community and health and social care services are to be ready when the new data start flowing, there is a need for experts across all the domains and disciplines relating to data access and sharing, including study participants and users of health, social care and other services, to have worked through all of these issues. Human-mediated decision-making bodies will undoubtedly be central to ensuring the trustworthiness of the most sensitive of those data, and achievable, reasoned and responsible decisions about their use. The METADAC model we describe here provides a template for such future governance.

## Abbreviations

ALSPAC: Avon Longitudinal Study of Parents and Children
DAC: Data Access Committee
METADAC: Managing Ethico-social, Technical and Administrative issues in Data ACcess
NHS: National Health Service
PLS: Plain language summary
REC: Research Ethics Committee
TRT: Technical Review Team
UKDA: UK Data Archive
